# Effect of Dietary Carbohydrate-to-Protein Ratio on Gut Microbiota in Atlantic Salmon (*Salmo salar*)

**DOI:** 10.3390/ani9030089

**Published:** 2019-03-11

**Authors:** Alejandro Villasante, Carolina Ramírez, Natalia Catalán, Rafael Opazo, Patricio Dantagnan, Jaime Romero

**Affiliations:** 1 Laboratorio de Biotecnología de Alimentos, Unidad de Alimentos, Instituto de Nutrición y Tecnología de los Alimentos (INTA), Universidad de Chile, Santiago 7830490, Chile; alejandro.villasante@gmail.com (A.V.); carolina.ramirez.saavedra@gmail.com (C.R.); nataliabcatalant@gmail.com (N.C.); ropazo@inta.uchile.cl (R.O.); 2 Núcleo de Investigación en Producción Alimentaria, Facultad de Recursos Naturales, Departamento de Ciencias Agropecuarias y Acuícolas, Universidad Católica de Temuco, Temuco 4781312, Chile; dantagna@uct.cl

**Keywords:** Atlantic salmon (*Salmo salar*), microbiota, carbohydrates, carbohydrate/protein ratio

## Abstract

**Simple Summary:**

Carbohydrates, in the form of energy reserve polysaccharides, are major food components that supply low-cost energy in farm animal feed formulation. Most of these compounds are obtained from plant ingredients (i.e., cereal grains). As the aquaculture industry moves towards formulating marine-derived ingredients free diets, the inclusion of plant ingredients is expected to continuously increase, and thus the amount of carbohydrates in aquafeed formulation will increase as well. Carnivorous fish, including salmonids, show a slow blood glucose clearance rate and suboptimal growth performance when fed rich carbohydrate meals. The role of gut microbial communities on carbohydrate utilization has been poorly explored in salmonids. Hence, we conducted an experiment to evaluate the effect of feeding a high carbohydrate diet to Atlantic salmon (*Salmo salar*) on gut microbiota composition. Our results suggest increasing the level of digestible carbohydrate mostly affects low-abundance bacteria in favor of those capable of using carbohydrates as a major energy-yielding substrate. Further study for a better understanding of the role of gut microbiota in carbohydrate utilization in carnivorous fish is required.

**Abstract:**

Atlantic salmon (*Salmo salar*) is a carnivorous fish species whose productive performance tends to be suboptimal when fed low-cost carbohydrate rich meals. It is of interest to study the dynamics of gut microbiota communities in salmonids fed high carbohydrate diets since gut microbes are referred to as key players that influence the metabolism and physiology of the host. A study was conducted to determine the effect of feeding a high carbohydrate diet to Atlantic salmon in gut microbiota communities. A medium carbohydrate (15% wheat starch)/medium protein (MC/MP) diet or a high carbohydrate (30% wheat starch)/low protein (HC/LP) diet was fed to triplicate tanks (28 fish each) during four weeks. We conducted an in-depth characterization of the distal intestine digesta microbiota using high-throughput sequencing of the V4 region of the 16S rRNA gene. *Firmicutes*, *Actinobacteria* and *Proteobacteria* were the major phyla determined in either experimental group. Phylum *Planctomycetes*, class *Planctomycetia*, order *Planctomycetales* and genus *Lactococcus* were significantly more abundant in fish fed the HC/LP diet compared with fish fed the MC/MP diet. Our study suggests feeding a carbohydrate rich meal to salmon exerts a low impact on the structure of gut microbial communities, affecting mostly low-abundance bacteria capable of metabolizing anaerobically carbohydrates as a major energy-yielding substrate.

## 1. Introduction

In the last few decades, the aquafeed industry has gone through major changes in diet formulation to enhance fish performance in culture systems and to promote sustainability of the growing aquaculture industry [1,2,3]. Increasing inclusion of plant-derived ingredients in lieu of marine-derived feedstuff in finfish diets is part of the initiatives to reduce aquaculture’s dependence on marine resources. The use of plant-derived feedstuff has led to several challenges from a nutritional point of view, such as the increment of carbohydrate added in diet, in carnivorous fish farming. Carbohydrates are the primary component of grains, legumes and oilseeds, and they can be classified as either “energy reserve polysaccharides” (digestible carbohydrates) or “structural polysaccharides” (non-starch polysaccharides; NSPs) [4]. The inclusion of carbohydrates as a low-cost energy substrate reduces the overall feeding cost of fish production. Despite fish not having dietary carbohydrate requirements in a biological sense, optimal dietary inclusion of digestible carbohydrates evokes a protein and lipid sparing effect in fish. Feeding carbohydrates to fish increases protein and lipid retention for tissue growth and maintenance by preventing them from being catabolized [5]. Moreover, digestible carbohydrates provide metabolites for the synthesis of other compounds of biological importance in fish [6]. It is well-known that feeding habits, anatomical-physiological features and rearing conditions affects the ability to use digestible carbohydrates when farming fish [5]. The nutritional value of carbohydrates varies among fish species; for example, herbivorous and omnivorous species display a more efficient bioenergetics after being fed carbohydrate rich meals, due to greater amylase activity, intestinal glucose uptake capacity and control of glycaemia, compared to carnivorous fish. In addition, warm-water species are able to use more efficiently digestible carbohydrates compared to cold-water species, most likely due to the direct effect of temperature on kinetic of digestive and metabolic enzyme [5,6]. In the case of carnivorous fish, such as salmon and trout, evolution has shaped their anatomy, physiology and metabolism to efficiently perform at their trophic level. This means carnivorous fish satisfy their nutritional and energetic requirements from feeds with high contents of protein and lipid but low input of nutritive carbohydrates. Consequently, carnivorous fish are not adapted to efficiently use dietary carbohydrates, though they have a wide range of physiological and cellular mechanisms involved in glucose homeostasis regulation. An example of this poor ability to use dietary carbohydrates is a low intestinal glucose uptake capacity and reduced blood glucose clearance [5,7]. In carnivorous fish, most carbohydrate research has been conducted on nutrition and metabolism, homeostasis and clearance of glucose; however, less attention has been addressed to gut microbiota ecology, and its potential role on carbohydrate digestion and metabolism. Furthermore, differences might be observed when comparing indigenous or autochthonous (resident) microorganisms with allochthonous (transient) microorganisms. Autochthonous microorganisms are native to a particular habitat, being independent of exterior organic matter and periodic increases of nutrients. On the other hand, allochthonous microorganisms depend on the occasional increase of nutrients or the presence of unusual substrates [8]. In this regard, Gatesoupe et al. [9] evaluated the effects of different sources of digestible carbohydrates on metabolic response and intestinal microbiota in sea bass (*Dicentrarchus labrax*) juveniles. The authors observed feeding starchy diets to fish induced changes in gut microbiota profile, and the effect appears to be different between resident microbiota (intestinal mucosal-associated microbiota) and transient microbiota (digesta microbiota) in fish fed the same diet. Further, Geurden et al. [10] observed feeding a high digestible carbohydrate/low protein ratio diet (40% gelatinized starch + 20% glucose) to rainbow trout (*Oncorhynchus mykiss*) alevines modulates intestinal fungi profile in juveniles. However, to our knowledge, no study has been conducted to evaluate the effect of digestible carbohydrate on gut microbiota in Atlantic salmon (*Salmo salar*). Therefore, we conducted a study to determine the effect of feeding a high carbohydrate/protein ratio to Atlantic salmon in a 4-week period, using in depth-gut microbiota analysis. This is a powerful approach based on high-throughput sequencing of the 16S rRNA gene to study the composition of intestinal microbial communities, including uncultivable organism by using traditional methodologies (i.e., biochemical and phenotypic characteristics of bacteria) [11].

## 2. Materials and Method

### 2.1. Fish and Rearing Conditions

The study was conducted in the freshwater recirculation system at the Instituto de Nutrición y Tecnología de los Alimentos (INTA) of Universidad de Chile. Fish management protocol was in line with the recommendations of Guide for the Care and Use of Laboratory Animals of the National Institutes of Health, and the Committee on the Ethics of Animal Experiments of INTA, Universidad de Chile. A total of 168 fish (*Salmo salar*; 80 ± 10 g) were obtained from a commercial freshwater fish farm (Piscícola Huililco, Pucón, IX Región, Chile). Fish were randomly distributed in six 150-L fiberglass tanks (100-L work volume) supplied with well-aerated fresh water at a constant temperature (15.5 ± 0.5 °C; 8 L min^−1^; >90% oxygen saturation), under a 12 h light/12 h dark photoperiod. Fish were acclimated to experimental conditions and fed a medium carbohydrate/medium protein diet (MC/MP; with 15% of wheat starch, 50.1% of crude protein and 16.2% of fat) during a four-week period. The daily freshwater replacement rate was close to 50% in the recirculation system. Water quality physiochemical parameters (i.e., oxygen, temperature, pH and nitrate concentration) were checked on a daily basis.

### 2.2. Feeding Trial

Two experimental diets fulfilling the National Research Council nutritional requirements of *Salmo salar* [3] were produced by extrusion cooking with a laboratory twin-screw extruder (CLEXTRAL BC-21, Clextral, Firminy, France) at the Feed Pilot Plant of the Universidad Católica de Temuco, Temuco, IX Región, Chile. The experimental diets were a medium carbohydrate/medium protein diet (MC/MP; with 15% of wheat starch) and a high carbohydrate/low protein diet (HC/LP; with 30% wheat starch). Experimental diets were formulated considering a dietary protein background based on a mixture of fishmeal and plant meal blend to be isolipidic and isoenergetic (Table 1). Either experimental diet was fed to triplicate tanks (28 fish per tank) for a trial period of 4 weeks. Fish were fed by hand three times per day to apparent satiation, six day per week during the experimental trial. After 48 h of feed deprivation, fish were bulk weighed in each tank at the beginning and end of the trial.

### 2.3. Sampling Procedure

During the trial and after 48 postprandially, two fish per tank (*n* = 6) were randomly selected for determination of hepatosomatic and viscerosomatic index at the beginning and at the end of the trial. Briefly, fish were euthanized with an overdose (200 mg L^−1^) of tricaine methanesulfonate (MS222; Argent Chemical Laboratories, Redmond, WA, USA); individual weight and length were measured, following by liver and viscera weight determination. Additionally, at the end of the trial and after 6 h postprandially, a total of 11 samples of distal intestine contents were randomly sampled; six fish fed the MC/MP diet (*n* = 6) and five fish fed the HC/LP diet (*n* = 5). Similarly, fish were euthanized, and abdominal incision was performed to aseptically remove the distal intestine. The distal intestine segment was aseptically dissected and digesta collected in a 1.5 ml eppendorf tube. The digesta samples were flash frozen in liquid nitrogen, and stored at −80 °C until DNA extraction to perform the microbiota analysis. Microbiota analysis was conducted on intestinal digesta instead of fish stool to avoid contamination with environmental microorganism (i.e., tank water bacteria), which could have biased the results.

### 2.4. DNA Extraction and Sequencing

DNA was extracted from distal intestinal digesta lysed homogenates (0.25 g) using the MOBIO PowerFecal^®^DNA Isolation Kit (MO BIO Laboratories, Carlsbad, CA, USA) according to the manufacturer’s protocol. DNA concentrations were determined using the Qubit® dsDNA HS Assay kit (Life Technologies, Grand Island, NY, USA). The V4 region of the 16S rRNA gene was amplified following the fusion primer method using the primers 515F (GTGCCAGCMGCCGCGGTAA) and 806R (GGACTACHVGGGTWTCTAAT) as described by Caporaso et al. [12]. Variable region 4 was selected because of its high coverage, low error rate and its minimal loss of taxonomic resolution [13,14]. The resulting amplicons were of suitable length to be used in the Illumina^®^ Inc. sequencing platform. All PCR reactions were performed in duplicates in a 30 µL reaction mixture containing 1.5 U (5U/µL) GoTaq^®^ G2 Flexi DNA Polymerase (Promega, Madison, WI, USA), 6 µL of Buffer (5×), 2.4 µL of Mg (25 mM), 1.2 µL of nucleotides mix (5mM each), 0.3 µL of primers (20 µM) and 18.5 µL of nuclease free water. In addition, a negative PCR control without the DNA template was run. The PCR conditions included an initial denaturation at 94 °C for 5 min, followed by 35 cycles of denaturation at 94 °C for 30 s, annealing at 56 °C for 30 s, and extension at 68 °C for 45 s. After the cycling procedure, the amplicons from each sample were pooled and run on a 1% agarose gel. Subsequently, the amplicons were purified with the QIAquick® PCR Purification kit (Qiagen, Germantown, MD, USA,). Libraries were sequenced on the paired end Illumina platform Hiseq PE250 adapted for 300-bp paired-end reads at the CD Genomics (http://www.cd-genomics.com).

### 2.5. Bioinformatics Analysis

For microbial communities analysis, we assembled quality-filtered reads into error-corrected amplicon sequence variants (ASVs) using Devisive Amplicon Denoising Algorithm (DADA2) v1.6.0 microbiome pipeline (available at https://github.com/benjjneb/dada2) to identify the presence and abundance of different microbial taxa based on the assembly of the 16S rRNA sequence reads. This microbiome pipeline describes microbial communities using unique ASVs, which represent unique bacterial taxa [15]. Using read quality scores for the dataset, forward and reverse reads were truncated at 285 bp and 275 bp, respectively; using the filterAndTrim function with standard parameters (maxN = 0, truncQ = 2, and maxEE = 2). DADA2’s error model automatically filters out singletons, removing them before the subsequent sample inference step. Sample inference was performed using the inferred error model and chimeric sequences were removed using the removeBimeraDenovo function. Assembled ASVs were assigned taxonomy (phylum to genus) using the Ribosomal Database Project (RDP) naïve Bayesian classifier (implemented in DADA2) and the “RDP training set 14” [16]. Using the R package Phyloseq [17], we eliminated any ASV without a bacterial phylum assignment, and also those assigned to Cyanobacteria/Chloroplast. Using DADA2, no rarefying of sequence reads was necessary. Finally, Illumina next-generation DNA sequences were deposited in the Sequencing Read Archive (SRA) of the National Centre for Biotechnology Information under Bioproject accession PRJNA498084, SRA run accessions SRX4924180-SRX4924190.

### 2.6. Ethical Notes

The study was conducted in accordance with the guidelines of the Bioethics and Biosecurity Committee of the Instituto de Nutrición and Tecnología de los Alimentos (INTA) at Universidad de Chile. The ethical approval code is FCYT2/17JR.

### 2.7. Statistical Analysis

All statistical analyses were performed using “R” v. 3.4.3 (http://www.R-project.org). Fish growth parameters were analyzed for normality (Kolmogorov–Smirnov test) and homoscedasticity (Levene’s test) following a Student’s *t*-test with a 5% of significance level. In the case of detecting non-equal variance, the non-parametric Wilcoxon-Mann-Whitney rank sum test was conducted. For microbiota data, analyses were performed in “R” with accompanying packages Phyloseq and Vegan [18]. Alpha diversity measured by the Shannon and Simpson diversity index and species richness measured by Chao1 was calculated using Phyloseq [17]. The normality was tested with the Shapiro-Wilk test. Unweighted and Weighted UniFrac distances were calculated as β diversity measures using the phyloseq package in R [17]. To statistically test diet effects on the homogeneity of microbial community composition, we performed permutational multivariate analysis of variance (PERMANOVA) using package Vegan analyses on distance metrics. Differential taxa abundance was performed using LefSe [19]. This method involves the Kruskal-Wallis (KW) sum-rank test between classes of data followed by (unpaired) Wilcoxon rank-sum test to conduct pairwise tests among subclasses. LDA is then used to estimate the effect size for each of the identified taxa. We used LEfSe (Galaxy Version 1.0,) with default parameters (KW = 0.05; Wilcoxon = 0.05; LDA score threshold = 2.0) as well as using the all-against-all strategy for multi-class analysis. All other comparisons were made using either Welch’s *t*-test or Kruskal-Wallis (KW).

## 3. Results

### 3.1. Fish Growth Performance

After four weeks of feeding experimental diets to Atlantic salmon, mean final weights of fish were not statistically different between experimental groups (Table 2). Mean weight gain (g/fish) decreased with greater inclusion of digestible carbohydrate (wheat starch) in the diet. Furthermore, mean feed intake (g as fed/fish) showed no statistical significant differences among experimental groups. The feed conversion ratio (FCR) was significantly greater in fish fed the high carbohydrate/low protein diet compared with fish fed the medium the carbohydrate/medium protein diet. Daily growth coefficient (DGC) was significantly greater in fish fed the medium carbohydrate/medium protein diet compared with fish fed high carbohydrate/low protein diet. Finally, no significant statistical differences were detected in the hepatosomatic index (HIS) and viscerosomatic index (VSI) between experimental groups (Table 2).

### 3.2. High-Throughput Sequence Data

A total of 1.068.308 initial raw reads were obtained. After filtering by removing low-quality reads and chimeras, 389.196 reads were retained; 192,377 reads from MC/MP with an average of 38,475 ± 4505 reads per individual sample, and 196,819 reads from HC/LP with an average of 32,803 ± 6509 reads per individual sample. The number of total reads per sample is shown in Supplementary Material. Finally, after the elimination of the sequences not assigned to the phylum level and those assigned as cyanobacteria, a total of 484 ASVs were detected in all samples.

### 3.3. Diversity Analysis of Microbiota

**Figure 1:**
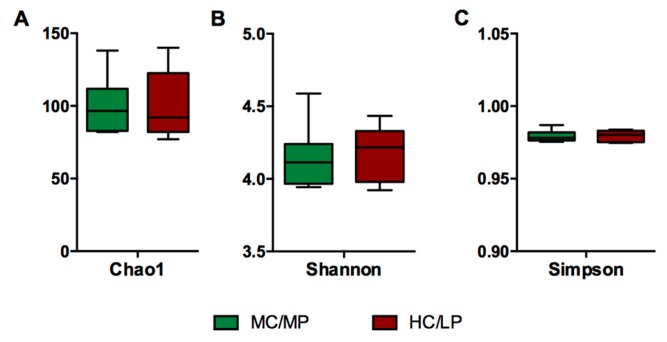
Representation of alpha diversity indexes of Atlantic salmon (*Salmon salar*) fed either medium carbohydrate/medium protein diet (MC/MP) or high carbohydrate/low protein diet (HC/LP). Diversity in the gut bacterial community was measured using Chao-1 (**A**), Shannon index (**B**), Simpson index (**C**). Chao-1 was evaluated using *t*-test. Shannon and Simpson index were evaluated using Mann-Whitney.

**Figure 2:**
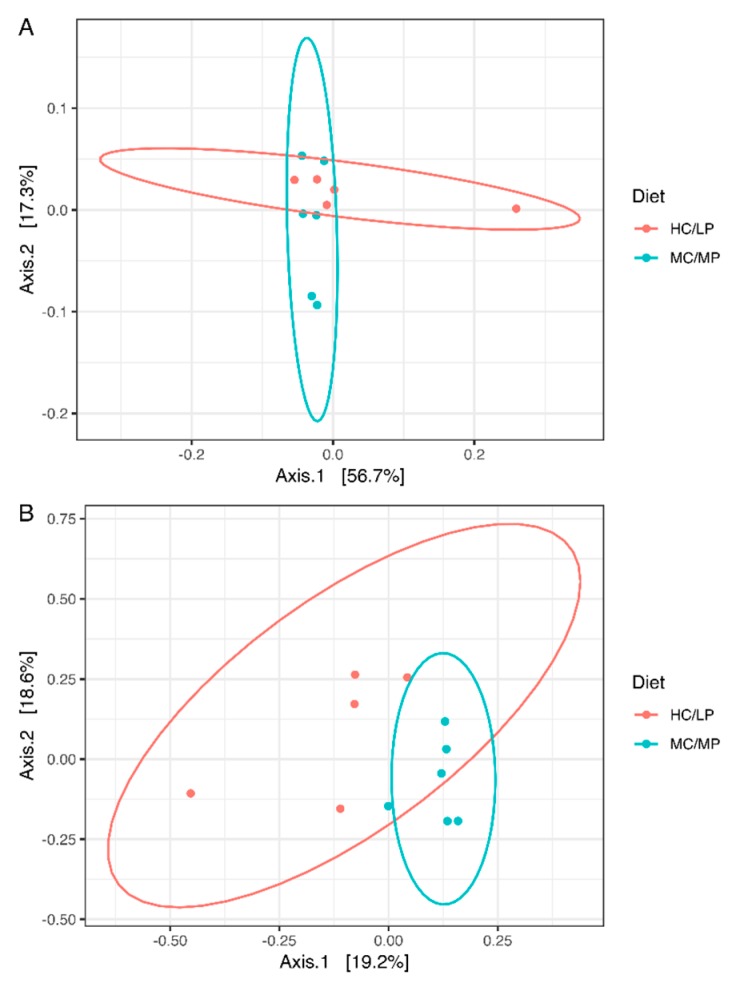
Principal coordinates analysis (PCoA) of the bacterial communities derived from the unweighted (**A**) and weigthed (**B**) UniFrac distance matrix. Circles represent individual samples from *Salmo salar* distal intestine digesta microbiota. Light blue circles correspond to samples derived from fish (*n* = 6) fed the medium carbohydrate/medium protein diet (MC/MP), and red circles correspond to samples from fish (*n* = 5) fed the high carbohydrate/low protein diet (HC/LP).

No significant differences in richness and either alpha diversity index between experimental groups were observed (Figure 1). Regarding beta-diversity, the unweighted UniFrac analyses revealed a significant difference (*p* = 0.007) of the bacterial communities of distal intestine digesta between fish fed the MC/MP diet and fish fed the HC/LP diet (Table 3). The principal coordinates plots were used to illustrate similarity in digesta-associated bacterial communities from fish fed either experimental diet, based on unweighted and weighted UniFrac analyses (Figure 2A,B, respectively). The two main components explained 74% of the total variance in the unweighted UniFrac analyses-derived principal coordinates plot (axis 1, 56.7%; axis 2, 17.3%). On the other hand, in the weighted UniFrac analyses, the two main components explained 37.8% of the total variance observed (axis 1, 19.2%; axis 2, 18.6%). Furthermore, we observed an outlier from the rest of the members of the HC/LP group, which means this individual showed dissimilarity when comparing with the other members of the same group (HC/LP), following the criteria of presence or absence of bacteria. That means this individual is more dissimilar with the rest of the group members in terms of low abundance bacteria.

### 3.4. Taxonomic Composition and Differential Abundance of Bacterial Communities of Distal Intestine Digesta of Fish Fed Experimental Diets p

**Figure 3:**
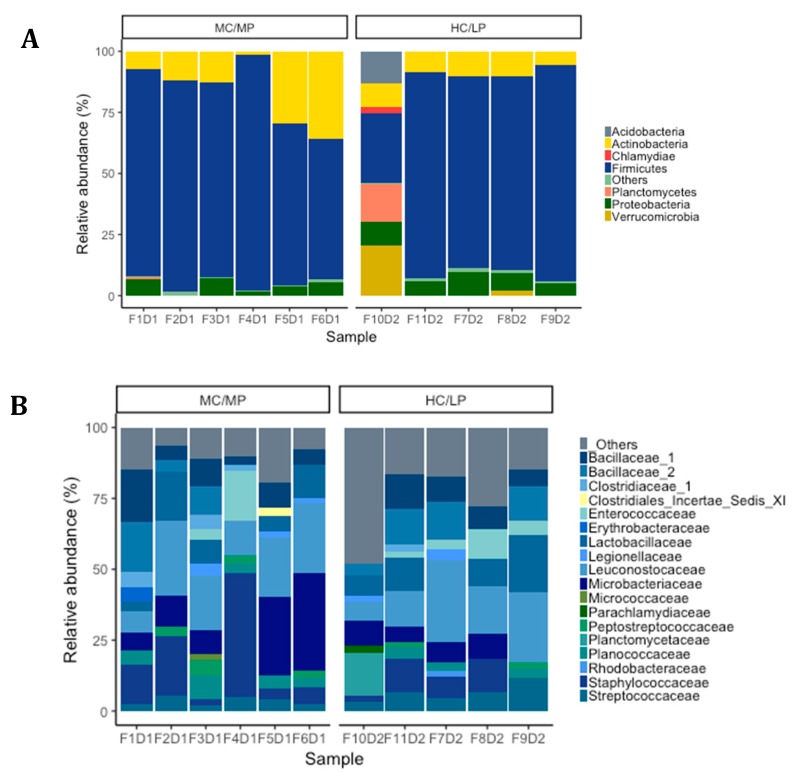

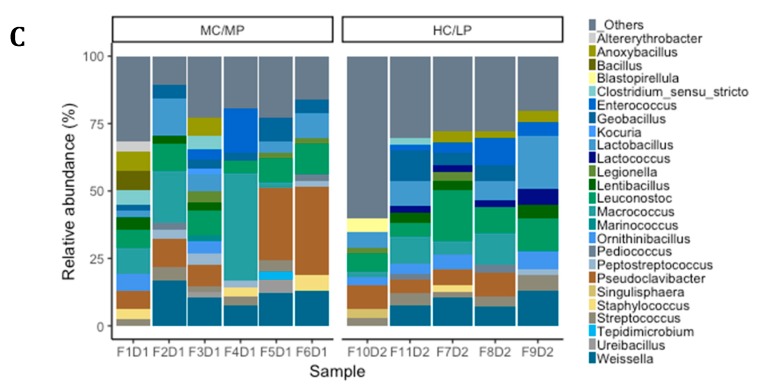
Relative abundance (%) at phylum level (**A**), family level (**B**) and genus level (**C**) for each sample in distal intestine digesta microbiota from fish (*n* = 6; F1D1, F2D1, F3D1, F4D1 and F5D1) fed the medium carbohydrate/medium protein diet (MC/MP) and from fish (*n* = 5; F10D2, F11D2, F7D2, F8D2 and F9D2) fed the high carbohydrate/low protein diet (HC/LP).

**Figure 4:**
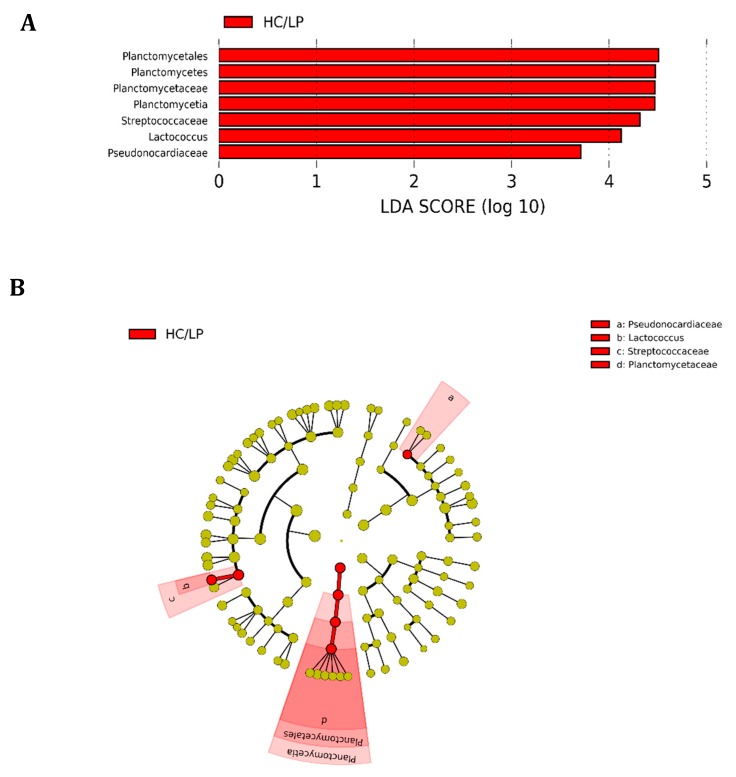
Differences in the distal intestine digesta microbiota of Atlantic salmon (*Salmon salar*) fed medium the carbohydrate/medium protein diet (MC/MP) compared with Atlantic salmon fed the high carbohydrate/low protein diet (HC/LP). Analysis of 16S rRNA reveals significantly greater relative abundance at different taxon levels in salmon fed HC/LP. (**A**) LDA score of abundance of taxa; (**B**) cladogram showing differentially abundant taxa (phylum to genus) of the distal intestine digesta microbiota of Atlantic salmon fed either experimental diet.

We observed *Firmicutes* (78.6%), *Actinobacteria* (16.5%) and *Proteobacteria* (4.2%) were the most abundant phyla in distal intestine digesta of fish fed the MC/MP diet. Similarly, in fish fed the HC/LP diet, the most abundant phyla were *Firmicutes* (71.8%), *Actinobacteria* (8.9%) and *Proteobacteria* (7.4%) (Figure 3A). At the family level, *Leuconostocaceae* (18.6%), *Staphylococcaceae* (15%), *Microbacteriaceae* (14.8%), *Bacillaceae_1* (8.4%), *Lactobacillaceae* (7.9%), *Bacillaceae_2* (5.8%), *Planococcaceae* (4.2%) and *Streptococcaceae* (*3.7%*), were the most abundant families in distal intestine digesta of fish fed the MC/MP diet. On the other hand, *Leuconostocaceae* (18%), *Lactobacillaceae* (10%), *Bacillaceae_2* (8.5%), *Bacillaceae_1* (7.1%), *Staphylococcaceae* (6.7%), *Streptococcaceae* (*6.6%*), *Microbacteriaceae* (6.4%) and *Enterococcaceae* (4.2%), were the most abundant families in distal intestine digesta of fish fed the HC/LP diet (Figure 3B). Finally, at the genus level, *Pseudoclavibacter* (14.3%), *Macrococcus* (12.2%), *Weissella* (10.1%), *Leuconostoc* (8.4%), *Lactobacillus* (5.3%), *Streptococcus* (3.1%) and *Staphylococcus* (2.7%), were the most abundant genera in fish fed the MC/MP diet. In fish fed the HC/LP diet, the most abundant genera were *Leuconostoc* (10.7%), *Lactobacillus* (8%), *Weissella* (6.1%), *Pseudoclavibacter* (5%), *Macrococcus* (4.2%) and *Streptococcus* (3.6%) (Figure 3C,B). The *Firmicutes/Proteobacteria* ratio was higher in the digesta of fish fed the MC/MP diet compared with fish fed the HC/LP diet (18.7 and 9.7, respectively). Significant differences in the relative abundance (LDA effect side score ≥ than 3.5; Figure 4A) at different taxon levels (using phylum to genus-level data) between fish fed either experimental diet were detected when performing LEfSe analysis. These differences are represented in a cladogram in Figure 4B. The phylum *Planctomycetes* showed significantly greater abundance in fish fed the HC/LP diet compared with fish fed the MC/MP diet. Similarly, the class *Planctomycetia* and the order *Planctomycetales* were significantly more abundant in fish fed the HC/LP diet. Finally, families *Planctomycetaceae, Streptococcaceae* and *Pseudonocardiaceae* as well as genus *Lactococcus*, were significantly more abundant in fish fed the HC/LP diet compared with fish fed the MC/MP diet.

### 3.5. Core Microbiome for Distal Intestine Digesta of Fish Fed Experimental Diets

**Figure 5:**
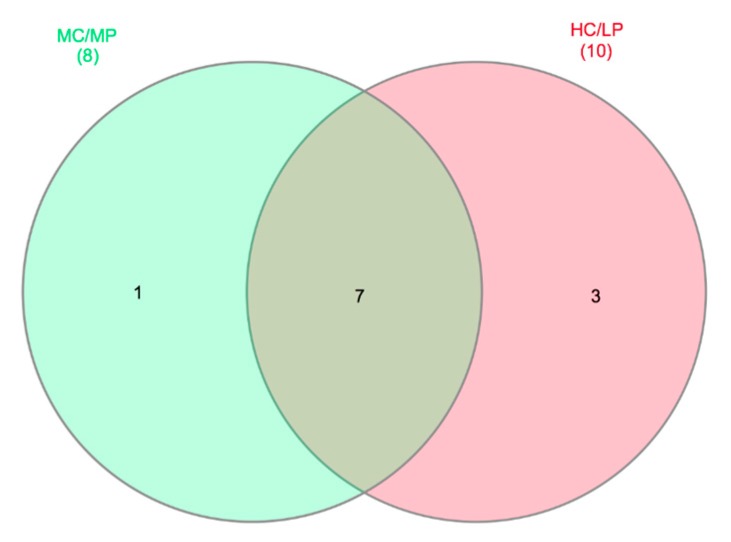
Venn diagrams showing distal intestine digesta core microbiota genera distribution. Seven genera were identified as core microbiota (80% of samples in each experimental group) in distal intestine digesta microbiota.

A core microbiome of seven genera, including *Macrococcus*, *Weissella*, *Leuconostoc*, *Streptococcus*, *Geobacillus*, *Lactobacillus* and *Pseudoclavibacter*, were detected for distal intestine digesta of fish fed either experimental diet (present at least in 80% of samples in each dietary treatment, Figure 5). *Staphylococcus* was the only genus exclusive for distal intestine digesta of fish fed the MC/MP diet. On the other hand, three genera including *Enterococcus*, *Lactococcus* and *Ornithinibacillus*, were exclusive for distal intestine digesta of fish fed the HC/LP diet.

## 4. Discussion

In fish, the capacity to use dietary carbohydrate is modulated by the interaction of several factors, including those from biological, dietary and environmental origin. Similarly to other livestock species, carnivorous fish species, such as salmon and trout, have physiological and cellular mechanisms, including digestive and metabolic enzymes, glucose transporters, hormones and glucose sensing components, to use glucose as energy yielding substrate. Notwithstanding the aforementioned fish, carnivorous fish, such as salmonids species, are considered to be “glucose-intolerant”, since they tend to show slow glucose turnover and prolonged postprandial hyperglycemia, after being fed digestible carbohydrate rich meals [5]. Although glucose regulation-associated mechanisms in farmed carnivorous fish has been discussed in numerous studies, the actual reason underlying the poor ability of carnivorous fish to use dietary carbohydrates as a major energy substrate, remains less clear [20]. One such aspect that has received less attention is the potential implication of gut microbiota in carbohydrate nutrition and metabolism in carnivorous fish fed digestible carbohydrate rich meals. To our knowledge, the present study reports the first in-depth characterization of gut microbiota in Atlantic salmon fed a high carbohydrate diet during a four-week trial.

Our results indicate differences in microbiota communities between fish fed MC/MP diet (15% starch) and fish fed HC/LP diet (30% starch), when considering the presence or absence (unweighted UniFrac) of bacteria in distal intestine digesta. However, when considering abundance of observed bacteria (weighted UniFrac), no differences in microbial communities between experimental groups were observed. The unweighted UniFrac analysis is more sensitive to detecting differences in low-abundance features. In contrast, weighted UniFrac analysis incorporates the number of reads assigned to an ASV, calculating shared/unshared branch lengths to compute distance between microbial communities from different origins, and thus diminishing the impact of low-abundance features [21]. Therefore, it appears that increasing the level of digestible carbohydrate mostly affects low-abundance microorganism with no changes in species richness between distal intestine digesta-associated microbial communities of salmon in the short term (Figure 1). In line with this statement, Geurden and colleagues [10] observed a marginal effect on intestinal microbiota composition when feeding a high carbohydrate/protein ratio diet to alevines and juveniles rainbow trout. The authors detected no significant dissimilarities in bacterial profile and only significant differences in fungal profile. This marginal effect on bacterial communities appears to be true for digestible forms of carbohydrates (i.e., starch), since previous work reported feeding diets supplemented with mannan oligosaccharide (non-digestible carbohydrates) to rainbow trout, modulates intestinal microbial communities by reducing species richness [22]. The above-mentioned effect might be a consequence of the specific molecular structure of mannan oligosaccharide differing with the molecular structure of starch-like polysaccharides. In this regard, the presence of certain sugars in mannan oligosaccharide, such as mannose, might attach to bacterial surface, thus facilitating their intestinal evacuation via feces [23]. On the other hand, previous works have reported a significant effect of feeding different plant-derived protein ingredients to salmonids on beta diversity analysis of gut microbiota communities [11,24,25,26,27,28]. It appears that increasing digestible carbohydrate imparts a marginal effect on gut-associated bacteria communities compared with the effect caused by replacing the source of dietary protein in carnivorous fish. In this regard, we observed a core microbiota constituted by seven bacteria genera from a total of 11 bacteria genera observed in the experiment. This means that most parts (64%) of the bacteria genera identified in the distal intestine digesta belong to the core microbiota. On the other hand, Gajardo and colleagues [26], who evaluated the effect of using alternative protein sources on intestinal microbiota in Atlantic salmon, detected a core microbiota constituted by 42% of the total OTUs identified in all diet groups. Similarly, studies replacing fishmeal by alternative protein sources reported a reduced core microbiota of 3.9% [11], 4.8% [28] and 12.4% [27] in salmonids. Therefore, we suggest the core microbiota may be proportionally greater when modifying the level of digestible carbohydrates compared with changes in the source of protein (i.e., fishmeal replacement in carnivorous fish feed). Carnivorous species belongs to a high trophic level whose natural feed is rich in protein (>40%) and very poor in carbohydrates (<1%) [29], and thus natural selection pressure has shaped their capacity to better deal with changes in dietary protein source rather than changes in digestible carbohydrates. This is especially true, since both intestinal microbiota and the host should co-evolve to ensure a successful symbiotic association in time [30]. Thus, fish from different trophic levels exhibit differences in metabolic capacity of their gut microbiota. In line with this concept, Liu and colleagues [31] demonstrated that host’s trophic level shapes the structure and composition of gut microbiota, metabolic capacity and gut content enzyme activity. Indeed, the authors detected that cellulose-degrading bacteria were dominant in herbivorous fish, while protease-producing bacteria were dominant in carnivorous species. Moreover, the predictive metabolic function analysis (PICRUSt) revealed greater cellulase and amylase activities in herbivorous fish than in carnivorous species, while trypsin activity in carnivorous fish was greater than in herbivorous fish. This means that carnivorous fish are anatomically, physiologically and metabolically better adapted to digest and utilize protein (i.e., plant or animal-derived protein) rather than carbohydrates, as main energy-yielding substrate. With regard to taxonomic composition, increasing the level of digestible carbohydrates did not affect the ranking of the major bacteria phyla (*Firmicutes* > *Actinobacteria* > *Proteobacteria*) in microbial communities of the distal intestine digesta in Atlantic salmon. However, the starchy diet caused a decrease in the *Firmicutes/Proteobacteria* ratio. Interestingly, previous studies have reported a greater ratio when increasing the inclusion of plant protein ingredients in lieu of fishmeal in salmonids diets [11,24,32]. Thus, it appears that the response of fish gut microbiota differs based upon the type of macronutrient (i.e., protein, lipid or carbohydrate) under modification.

In our study, experimental diets were formulated with a mixture of fishmeal/plant-meal blend in a similar ratio (0.7 and 0.8 for MC/MP and HC/LP, respectively), as dietary protein source. Therefore, the reduction of the *Firmicutes*/*Proteobacteria* ratio is most likely the consequence of increasing the carbohydrate/protein ratio. Similarly to our study, previous works have reported *Firmicutes* as the most dominant phylum in carnivorous fish, such as salmonids [11,25,26,33,34,35,36]. However, this seems to be true for the case of carnivorous fish in aquaculture systems, since *Proteobacteria* has been reported to be the most dominant bacteria phylum in carnivorous fish captured from the wild, including Atlantic salmon [37], yellowtail kingfish (*Seriola lalandi*) [38] and fine flounder (*Paralichthys adspersus*) [39]. Furthermore, the phylum *Planctomycetes* was significantly more abundant in fish fed with the HC/LP diet compared with the MC/MP group (Figure 4A). *Planctomycetes* are a unique type of microorganism that initially were described as eukaryotes and later acknowledged as an altered type of Gram-negative bacteria [40]. Interestingly, phylogenetic and metabolic analyses conducted in heterotrophic *Planctomycetes* revealed that an anaerobic metabolism was present among all major *Planctomycetes* lineages [41]. The authors demonstrated that *Planctomycetes* use both fermentations of carbohydrates, such as sucrose and sulfur reduction, as putative mechanisms for growth and survival under anaerobic conditions. Therefore, the significant increase in the relative abundance of *Planctomycetes* in fish fed the HC/LP diet might be indicative of a response of these microbial communities towards an increase in digestible carbohydrates to be fermented under the prevailing anaerobic condition in intestine. Similarly, at the genus level, *Lactococcus* was significantly more abundant in fish fed the HC/LP diet. *Lactococcus* species are members of the Lactic Acid Bacteria (LAB), characterized as Gram-positive, usually non-motile, non-sporulating bacteria, which have been identified as components of the gut microbiota in fish [42]. These bacteria produce lactic acid as a major product of the fermentative metabolism of sugar. Therefore, our results suggest that an increase in digestible carbohydrate may have promoted *Lactococcus* growth and survival in fish that were fed the starchy diet. LAB have been generally considered as favorable bacteria due to their abilities to antagonize bacterial pathogens, the implications of the increment of *Lactococcus* spp. in growth and health of fish fed the HC/LP diet needs further exploration.

Finally, regarding growth performance, fish fed the HC/LP diet had lower weight gain and a lower daily growth coefficient compared with fish fed the MC/MP diet; however, this effect did not reduce the final weight. This most likely due to our study not being long enough to reflect an effect in time. Further, growth data demonstrated that salmon fed a 15% starch diet were more efficient compared with salmon fed a 30% starch diet, as revealed by a lower FCR and slightly greater PER. These results are consistent with previous studies reporting similar effect on growth performance of carnivorous fish, including salmonids species, fed increasing levels of digestible carbohydrates [6,43,44,45,46]. Therefore, our findings are in agreement with carbohydrate optimum recommended inclusion level (15–25%) for salmonids and cold marine fish species [3].

In conclusion, the present study demonstrates that feeding a carbohydrate rich diet to salmon in the short term causes low impact in gut microbial communities, and particularly affects low-abundance bacteria. From our findings, it seems that increasing the digestible carbohydrate promotes bacteria capable of metabolizing anaerobically carbohydrates as a major energy-yielding substrate in intestinal digesta. Long-term changes in diet alter metabolic characteristics of vertebrate gut microbiomes [47,48]. Changes of microbiomes enable the host to utilize diet components that were not used before. This in turn will provide opportunities for development of new aqua-feed useful for aquaculture. While the aquaculture industry continues to move towards producing marine-derived ingredients free diets, mainly by increasing the inclusion of low-cost plant ingredients, a concomitant increase in the carbohydrates fraction (i.e., energy reserve and structural polysaccharides) is expected in carnivorous fish feeds. Therefore, improving our understanding of the dynamic of fish gut microbiota toward these dietary changes as well as the implications of this microbial ecology modulation on carbohydrate utilization, will contribute to the well-being and sustainability of aquaculture worldwide.

## Figures and Tables

**Table 1:** Ingredients and chemical composition of the experimental diets used to feed Atlantic salmon over four weeks.

Ingredients (%, Diet)	Diet	
	MC/MP	HC/LP
Fish meal ^1^	30	21
Soy protein concentrate ^1^	30	23
Wheat gluten ^1^	7.5	7.5
Wheat starch, gelatinized ^1^	15	30
Fish oil ^1^	14	15
Vitamin C (35%) ^2^	0.2	0.2
Vitamin Premix ^2,3^	0.8	0.8
Mineral Premix ^2,4^	0.2	0.2
Choline chloride ^2^	0.3	0.3
Dicalcium phosphate ^2^	2	1.7
L-Methionine ^5^	0	0.2
Lysine ^6^	0	0.1
Chemical composition (%, DM)		
Moister	4.7	7
Crude protein	50.1	41.5
Fat	16.2	16.6
Ash	9.4	7.4
Fiber	1.9	1.6
Gross energy (MJ/kg)	20.7	20.5

^1^ Cargill Chile Ltda. Coronel, VIII Región, Chile; ^2^ Veterquímica S.A. Santiago, RM, Chile; ^3^ per kg dry diet: thiamin mononitrate, 62 mg; riboflavin, 71 mg; niacin, 294 mg; calcium pantothenate, 153 mg; pyridoxine hydrochloride, 50 mg; folic acid, 22 mg; vitamin B_12_, 0.08 mg; d-biotin, 0.8 mg; myoinositol, 176 mg; retinal acetate, 8818 IU; vitamin D_3_, 588 mg; α-tocopherol acetate, 670 mg; menadione sodium bisulfite complex, 37 mg; ^4^ per kg dry diet: KI, 1.9 mg; MnSO_4_·H_2_O, 75.8 mg; ZnSo_4_·7H_2_O, 132.0 mg; Na_2_SeO_3_, 0.88 mg; CoCl_3_·6 H_2_O, 4.0 mg; CuSO_4_·H_2_O, 11.8 mg; FeSO_4_·H_2_O, 298.5 mg; ^5^ M9625, Sigma-Aldrich, Santiago, RM, Chile; ^6^ L5501, Sigma-Aldrich, Santiago, RM, Chile.

**Table 2:** Growth parameters of Atlantic salmon fed either experimental diet during a four-week period ^a^.

	Experimental Diets				*p* Value
	MC/MP (15% Digestible Starch)		HC/LP (30% Digestible Starch)		
**Initial weight, g/fish ^1^**	103.8	0.5	105.5	1.5	>0.05
**Final weight, g/fish ^1^**	117.8	0.7	118.0	0.1	>0.05
**Weight gain, g/fish ^b1^**	14.0 *	1.2	11.7	0.2	0.033
**Feed intake as fed, g/fish ^c1^**	26.5	1.7	25.6	0.3	>0.05
**FCR ^d1^**	1.5	0.1	1.9 *	0.0	0.032
**DGC ^e1^**	1.1 *	0.1	0.9	0.0	0.024
**PER ^f2^**	1.2	0.0	1.1	0.0	<0.05
**Hepatosomatic Index (%) ^g1^**	1.2	0.1	1.5	0.2	>0.05
**Viscerosomatic Index (%) ^h1^**	6.5	0.6	6.7	0.7	>0.05

^a^ Mean values with their SEM for three tanks per group (*n* = 3) Mean values marked with * were significantly different between the experimental diets (*p* < 0.05); ^b^ Weight gain (g/fish) was estimated as (g mean final weight − g mean initial weight); ^c^ Feed intake was estimated as the total amount of ingested food (g as fed) divided by the number of fish; ^d^ Feed conversion ratio (FCR) was calculated as (feed intake/wet weight gain); ^e^ Daily growth coefficient (DGC) was determined as [(mean final weight^(1/3)^ − mean initial weight^(1/3)^)/number of days)] × 100; ^f^ Protein efficiency ratio (PER) was determined as live weight gain (g)/protein intake (g); ^g^ Hepatosomatic index was estimated as (100 × (liver weight/body weight)); ^h^ Viscerosomatic index was estimated as (100 × (viscera weight/body weight)); ^1^ Student’s *t*-test; ^2^ Wilcoxon-Mann-Whitney rank sum test.

**Table 3:** Comparison of similarities in microbiota composition associated with distal intestine digesta between Atlantic salmon fed either a medium carbohydrate/medium protein diet or a high carbohydrate diet, during a four week period.

	Statistical Test	Test Statistic	*p* Value
Unweighted UniFrac	PERMANOVA	1.965	0.007 *
Weighted UniFrac	PERMANOVA	1.238	0.219

* Indicates a rejection of the null hypothesis of no differences among groups (*p* < 0.01).

## Supplementary Materials

The following are available online at https://www.mdpi.com/2076-2615/9/3/89/s1.
