# Supplementary material for: Effect of Dietary Carbohydrate-to-Protein Ratio on Gut Microbiota in Atlantic Salmon (Salmo salar)
Source: Animals (Basel). 2019 Mar 11;9(3):89. doi: 10.3390/ani9030089 (PMC6466077; doi:10.3390/ani9030089)
Supplement: Supplementary file 1 [file animals-09-00089-s001.pdf]

Supplementary Materials

Table 1. Number of reads (no chimera) per sample.

ID Fish	Experimental Diet	Number of Sequences
F1D1	MC/MP	36.784
F2D1	MC/MP	40.814
F3D1	MC/MP	36.731
F4D1	MC/MP	33.142
F5D1	MC/MP	36.944
F6D1	MC/MP	41.457
F1D2	HC/HP	44.906
F2D2	HC/HP	27.609
F3D2	HC/HP	32.002
F4D2	HC/HP	23.528
F5D2	HC/HP	35.279
